# An optimization design proposal of automated guided vehicles for mixed type transportation in hospital environments

**DOI:** 10.1371/journal.pone.0177944

**Published:** 2017-05-31

**Authors:** Domingo González, Luis Romero, María del Mar Espinosa, Manuel Domínguez

**Affiliations:** Design Engineering Area – Universidad Nacional de Educación a Distancia (UNED), Madrid, Spain; Politecnico University of Bucharest, ROMANIA

## Abstract

**Aim:**

The aim of this paper is to present an optimization proposal in the automated guided vehicles design used in hospital logistics, as well as to analyze the impact of its implementation in a real environment.

**Method:**

This proposal is based on the design of those elements that would allow the vehicles to deliver an extra cart by the towing method. So, the proposal intention is to improve the productivity and the performance of the current vehicles by using a transportation method of combined carts.

**Results:**

The study has been developed following concurrent engineering premises from three different viewpoints. First, the sequence of operations has been described, and second, a proposal of design of the equipment has been undertaken. Finally, the impact of the proposal has been analyzed according to real data from the Hospital Universitario Rio Hortega in Valladolid (Spain). In this particular case, by the implementation of the analyzed proposal in the hospital a reduction of over 35% of the current time of use can be achieved. This result may allow adding new tasks to the vehicles, and according to this, both a new kind of vehicle and a specific module can be developed in order to get a better performance.

## Introduction

Automated Guided Vehicle Systems (AGVS) were invented in the 1950s, and have since developed into a tested means for organizing modern intralogistics. Although the automotive industry was initially the leader in adopting them, almost all industries have come to use AGVS in order to optimize material flows [1].

The use of automated-guided vehicles (AGV) is undergoing a great development in inner distribution tasks within hospital logistics [2]. Currently there are a large number of manufacturers who commercialize this kind of autonomous robots [3]. The models used share some similar lines of design, which have evolved from the early models to the current ones, but each of them presents its own features [4]. This paper is going to put forward a proposal according to the optimization design of these kinds of vehicles, so as to improve their output. Nevertheless, the aim of this stage is not to reach a definitive development of all the required elements to complete this proposal, but to serve as a basis for an analysis of the appropriateness of the measure. Once the proposed solution has been validated, there would follow a necessary specific development by means of the application of concurrent engineering principles. By doing this, each one of the affected disciplines can provide the required knowledge, in order to develop the necessary elements to achieve their common aim in their fields of action. The development of this work, in the case of cross-functional equipment, would be carried out according to principles of collaborative engineering and, from a common base; this would provide the necessary elements to complete the proposed project in a coordinated way.

**Fig 1:**
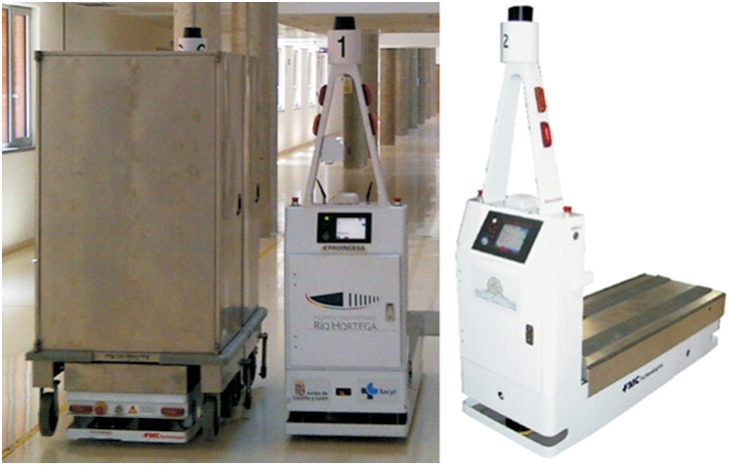
AGV L-shape, model Atlis.

The usual configuration of this kind of equipment consists of a wheeled base on which is incorporated a lifting platform to carry out the load and transport of carts used in the distribution of goods (Fig 1). On the front side it incorporates the control panel and the laser guided peak. The main common features of automated guided vehicles (AGVs), which are used in hospital logistics, are their L-shape design, as well as the method of carts’ transport by means of load. In order to carry out these loading operations, the robot is located under the cart and the platform is lifted, leaving the cart leaning on its base and ready to be transported [5]. During the unloading, the opposite operation is carried out: when the platform lowers, the cart is set free when it is leant on the floor. Afterwards the vehicle can continue doing the scheduled tasks, as it is possible to move the cart by hand to its final placement, which is facilitated by the wheels present on its base.

Therefore, the delivery is carried out cart by cart, spending a large part of operational time in the removal from the load points to the delivery ones. This means that a large part of time is spent facilitating shifts for a single service, as it is necessary to return to the load point after the delivery of every container. Furthermore, this problem is exacerbated due to the hospital’s current configuration, which has a large floor extension that forces it to cover distances of over 500 meters for a single service.

In the industrial field, the automated guided vehicles (AGVs) also use the towing method, in which a tractor unit pulls some carts or load platforms (tow type vehicles). These tow vehicles are usually released or loaded by hand, at the points of consumption in the reverse sequence of the walking direction, following a predefined route [6] [7]. Once all the carts are released, the sequence is carried out on the return path, and in that way the empty carts are united to the convoy. By means of the implementation of this system, the time spent in the shifts is shared between several carts, increasing the productivity, due to the optimization of the delivery routes. By contrast, the equipment’s maneuverability is lower, because they need more space in order to realize the turns. In addition, this system has a bigger problem in its implementation because, in the case of production centers with more than one floor, usage is not possible.

The proposal, which has been developed throughout this paper, involves the combination of both systems: the usual method of loading on the platform is complemented with the towing of an extra cart. The aim is to draw from a previous design of the needed elements and define the sequences of work, so that the mixed transport operations can be realized in an autonomous way, trying to get the advantages of both methods: that is, it combines the maneuverability in reduced spaces and their use in elevators with a reduction of time spent in the shifts. This is a novel system because, although both these systems are well developed separately, their automated mixed use is not so widespread, and much less so in the field of hospital logistics. The conclusions obtained from the analysis below will be used to determine the viability of the proposed measure and also to study their implementation in the vehicle’s design.

## An initial approach to the optimization´s proposal

There are many criteria, both from internal design engineering as well as from external ones, which must end up at the stage of design with the aim of developing optimization proposals for the equipment used in such a specific field. We have to take into account some external aspects which concern several branches of engineering science, whereas the internal aspects are those determinants linked to the product lifecycle and are therefore derived from the manufacturing process, the assembly, the maintenance or the recycling, following the principles of design engineering [8].

By contrast, the main external disciplines which must be taken into account in the design process, amongst others, are the architectural planning, the hospital logistical organization in the care services delivery models, the control and management systems or the robotics. The success of this project will depend to a great extent on the appropriate confluence of these different disciplines in the stages of design and development of the optimization proposals. The following sections will detail the basic aspects that configure this system according to the determining factors, which have been defined by the different disciplines mentioned previously, and which take part in the process. In order to do this, they have taken into account the gathered information as well as the experience from work conducted with this kind of equipment, mentioning the main constraints and their possible points of conflict.

### Basic description of the delivery system

The transport system that makes use of the automated guided vehicles (AGVs) used in hospital logistics, by means of standard carts, links the point of origin with the consumption ones in the destination units. In order to carry this out, some loading and unloading bays have been set up, where the containers to transport are parked; these communicate with robots and with the rest of the equipment that takes part in the delivery system (such as elevators, automatic doors…) by means of two different systems: radio-frequency identification (RFID) and presence detectors. The information provided by these systems interacts with the equipments’ configuration that—together with the scheduling—allow this equipment to have an autonomous operation according to predefined routes.

The containers’ loading and unloading operations are always carried out in the opposite way to the walking direction, by gathering and placing the cart in the origin and destination bays. These bays incorporate a magnetic side strip in order to set the cart’s exact position, and a presence detector informs the system as much as possible of the responsibilities of the later capillary distribution, about whether the container has been placed in that position or not.

**Fig 2:**
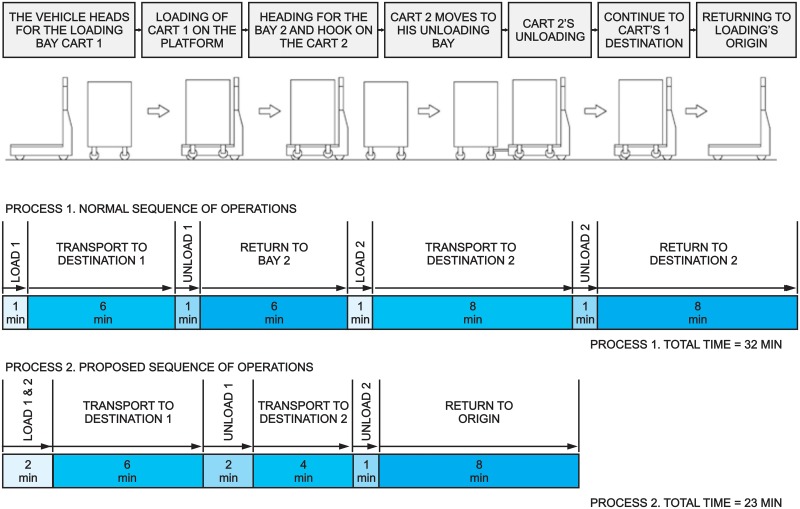
Sequence of proposed operations and a timing diagram.

In the case of the suggested proposal, which consists of a combined delivery, the basic operating practice would be to load a cart by means of a conventional system, and later it would be possible to hook on the cart that will be towed automatically. The unload would be made in the reverse sequence: in the first place the driven cart would be released in an unloading bay, which is equipped for this system, and then the cart that is transported by the vehicle’s platform would be unloaded. The implementation of this delivery system requires—as much in the case of vehicles as in the case of carts—some modifications which allow transport by means of towing but without affecting transport capacity due to vehicles’ load. Fig 2 shows a simplified diagram of a suggested system, together with a timing diagram that shows the possible impacts of the measure in a delivery operation. As illustrated, the reduction of time in a single operation can be 9 minutes out of 32 minutes total time, which means a reduction of around 30% of time spent.

### System prerequisites

**Fig 3:**
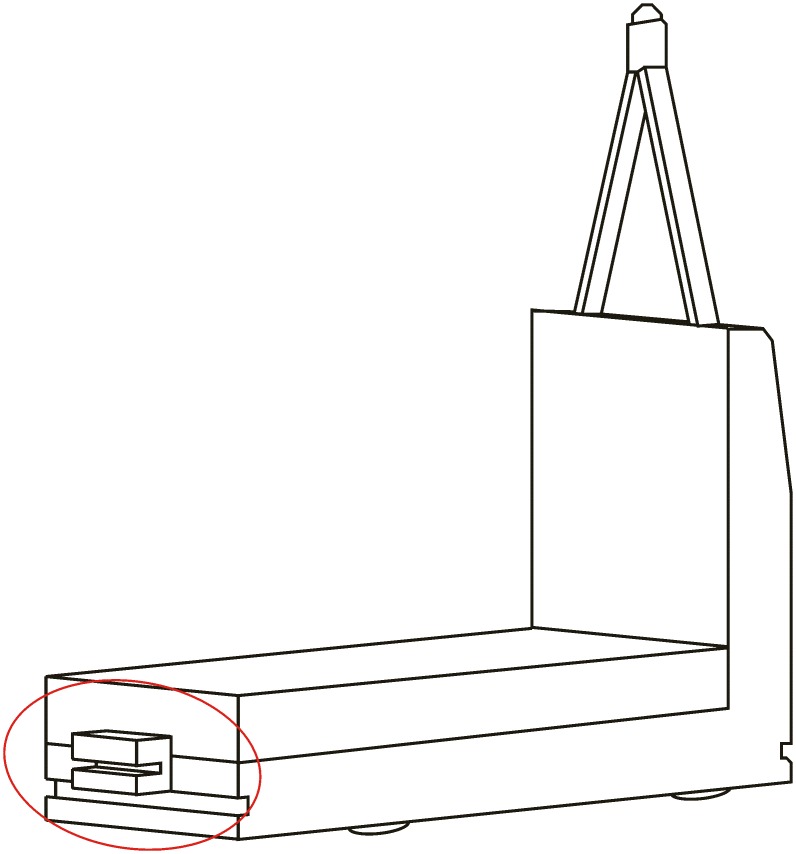
System prerequisites 1.

**Fig 4:**
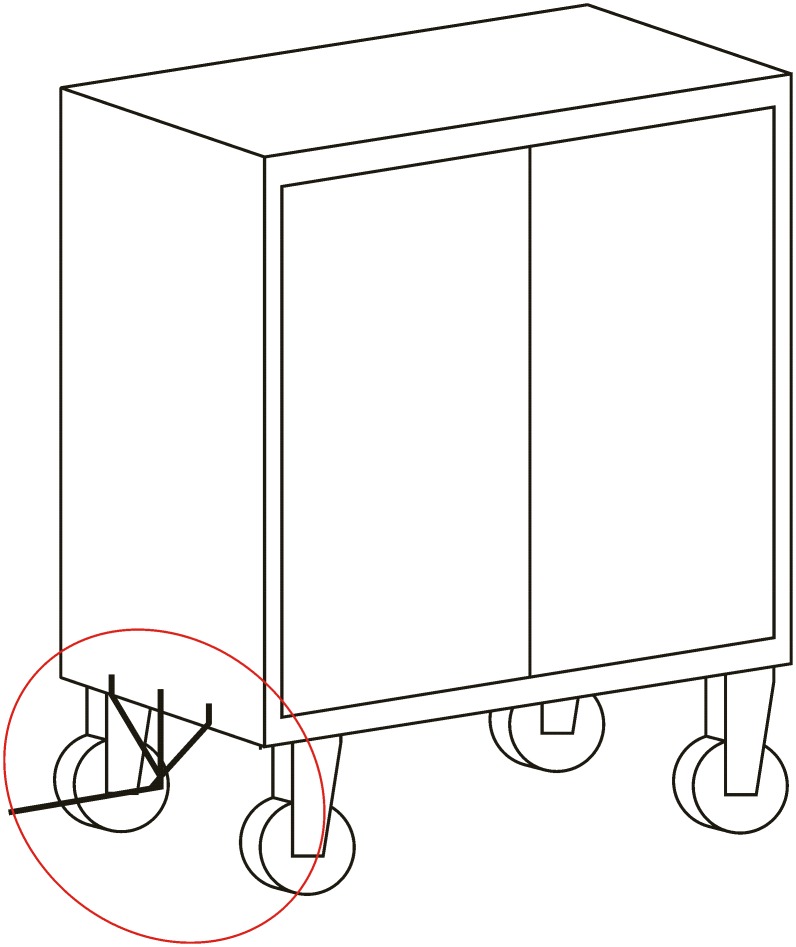
System prerequisites 2.

**Fig 5:**
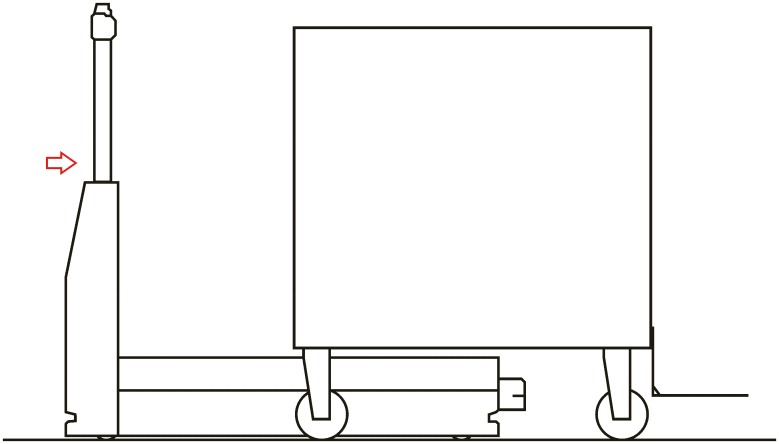
System prerequisites 3.

**Fig 6:**
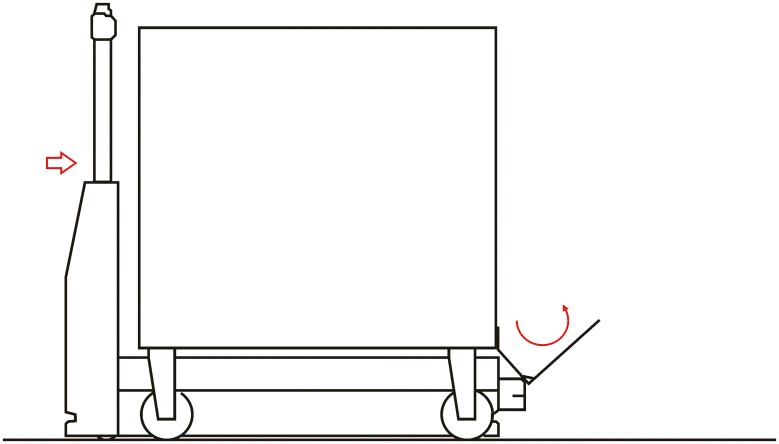
System prerequisites 4.

**Fig 7:**
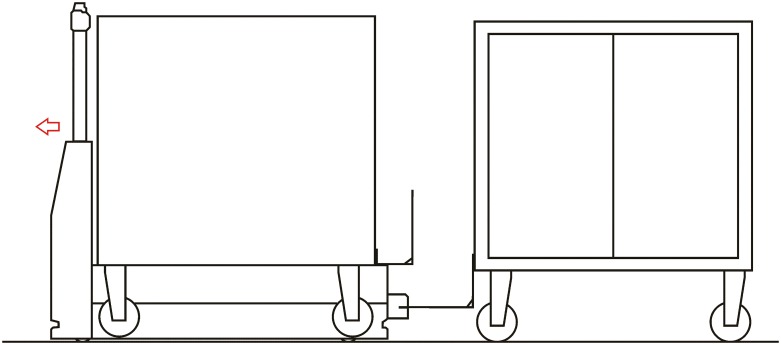
System prerequisites 5.

When the initial analysis of all the aspects required in order to develop the mixed type delivery is done, it is necessary to take into account those mandatory prerequisites in order to carry out their implementation. In that sense, there are many elements affected which take part in the development of the optimization proposal; the most important of them are mentioned below:

- **Automated hitch**. This is the key of the proposed system because it determines that the whole sequence of operations can be automated. By means of this mechanism, the vehicle can autonomously hook and release the cart that is towed. There are not many options to carry out this solution and the proposed solution, which has similar approaches in the industrial field, consists of the inclusion of an external unit that is incorporated in the existing equipment, and by means of doing this, the vehicle’s scope of action is extended considerably. Fig 3 shows a scheme about their possible positioning at the top of the vehicles.
- **Hitching point’s connection system with the cart**. This system must fulfill two main conditions: that it could be incorporated to existing carts and that it could allow the containers to be transported by using a load and tow system. That is, the system used for the hitch must not make conditional to the possibility that carts can be loaded onto the vehicle’s platform. Fig 4 shows one of the possible solutions for this, schematically, using a folding gravity system. Furthermore, in that way a single kind of cart can be used and thereby simplify the maintenance operations with the standardization of auxiliary resources. Figs 5, 6 and 7 show a schema about the loading operations’ sequence of both carts.
- **Loading bays used by towed carts**. By using the traditional method the carts are unloaded when the vehicle is negotiating the act of going backwards, therefore bays are restricted by vertical parameters. This unloading operation, which is carried out going backwards, is more complicated due to the fact that the vehicle transports a driven cart, so it seems simpler to do the unloading as a continuous process. That means that when the vehicle passes through the indicated area the hitch releases the cart, and this can continue to its next destination to carry out the unloading using the traditional method.
- **Equipment’s configuration**. The equipment’s control system must allow the required modifications to implement this system. Nowadays, Radio-Frequency Identification (RFID) gives the information about origin and destination but, with the implementation of the new system, many origins and destinations are doubled. Therefore, the software architecture must allow the implementation of this transport system. At the beginning, this aspect can be the one which raises the most problems due to, in many cases, the control systems being closed, and these modifications would require the equipment manufacturer’s direct intervention [9] [10].
- **Route’s design**. Previously, it was necessary to check that the route’s design had been defined to allow the cart’s transport to maneuver with a towing cart. Because of the bigger size of the unit, it is possible that some adjustments might become necessary in the case of the route’s design being included in the rescheduling of the guided system [11] [12].

### Operations sequence

Once the elements that take part in the process have been defined, and it has been noted that the system can be implemented, the next step is to analyze the different operations that are required in the maintenance logistics tasks. Below, the process and the operations sequence are detailed and shown graphically, because there are as many loading processes as unloading processes that have to be systematized in the task of transportation combined carts.

**Fig 8:**
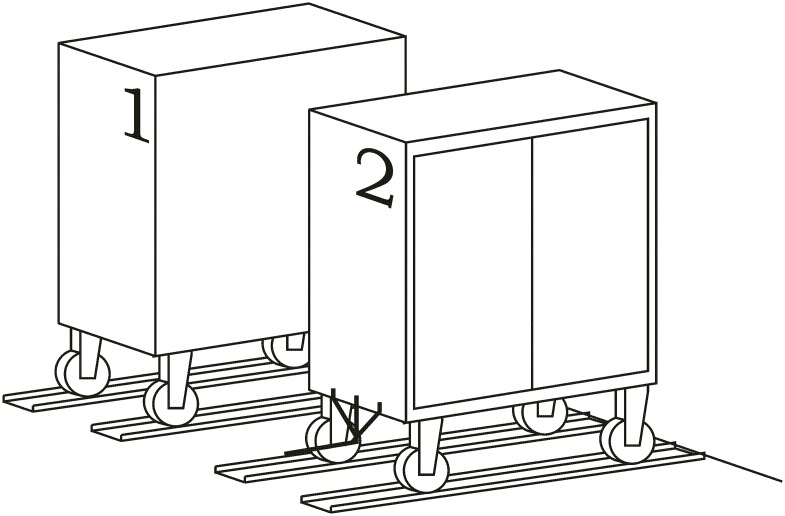
Loading operations 1.

**Fig 9:**
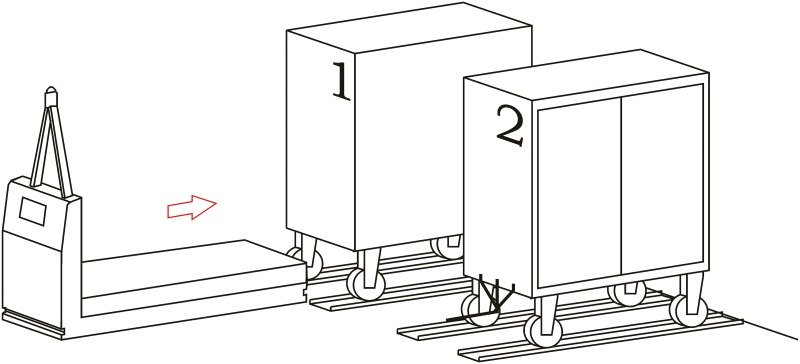
Loading operations 2.

**Fig 10:**
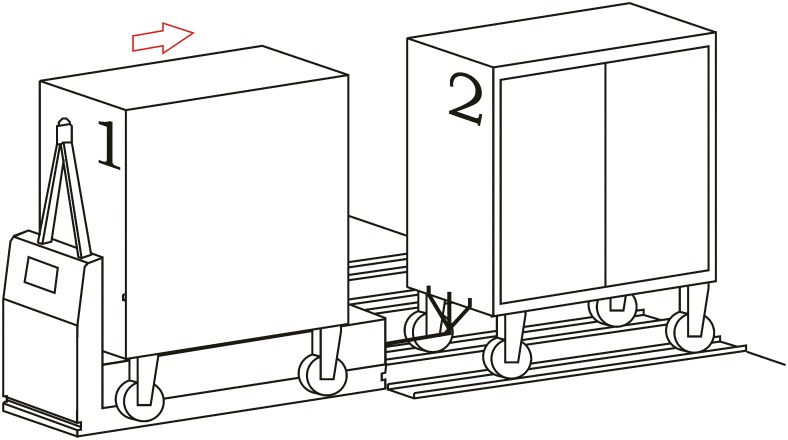
Loading operations 3.

**Fig 11:**
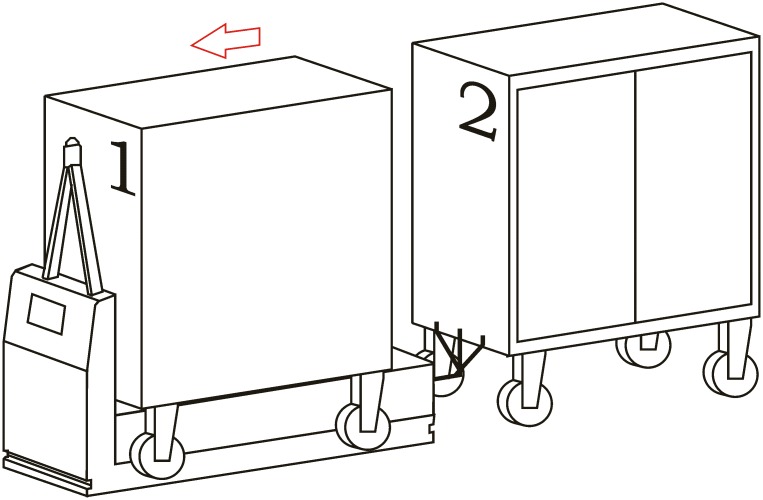
Loading operations 4.

**Fig 12:**
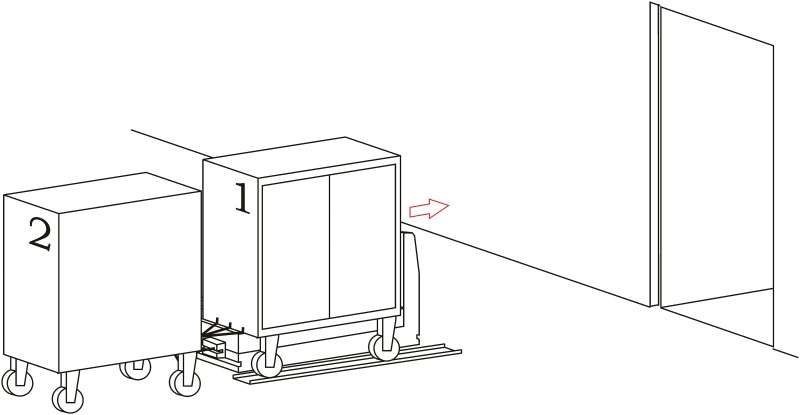
Unloading operations 1.

**Fig 13:**
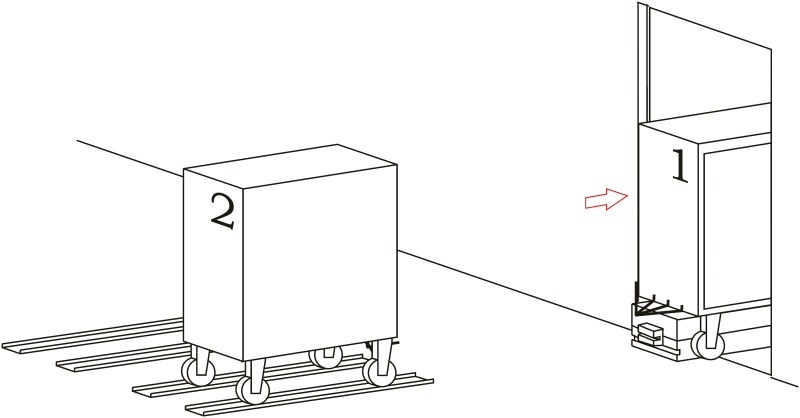
Unloading operations 2.

**Fig 14:**
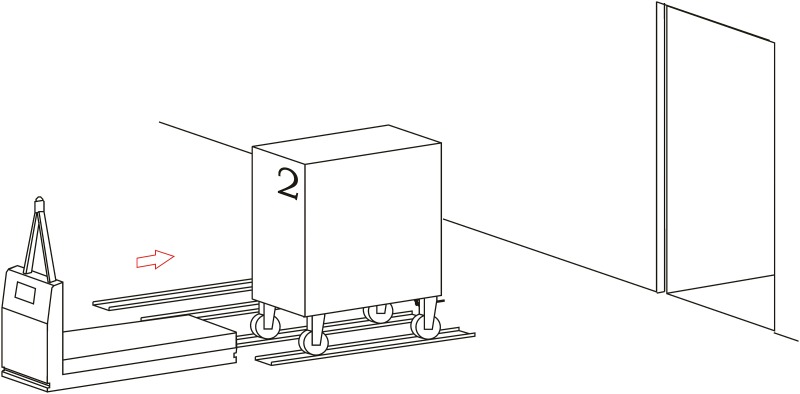
Unloading operations 3.

**Fig 15:**
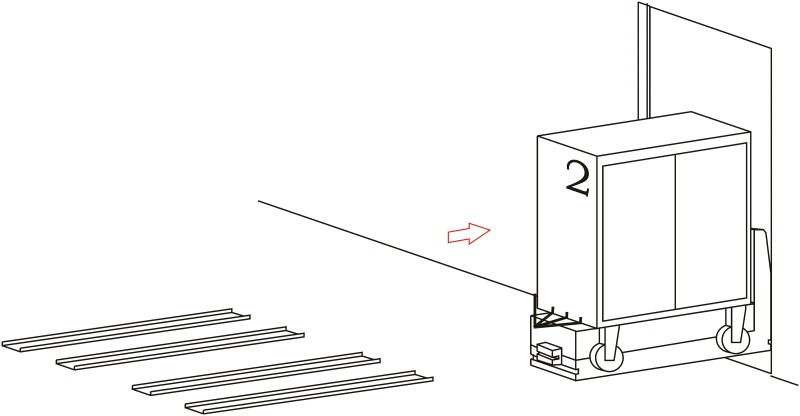
Unloading operations 4.

- **Loading operation**. In the first place, carts are parked in their assigned loading bays (Fig 8). Cart 1, which is transported on the vehicle’s platform, must be placed with the hook system on the inner side. The vehicle loads Cart 1 using a traditional method (Fig 9) and later the automatic hitch of Cart 2 is carried out (Fig 10). Both operations are carried out in the opposite way to the walking direction. Finally, the unit is ready in the position of being transported to the loading point, where it is placed with the cart that is loaded in second place (Fig 11).
  The loading operations in the case of returning empty containers, or containers loaded with materials that have not been used yet, are the same as in their origin, with the proviso that it is possible that the unloading bays are not close together. In that case, the priority is always the loading of the first container, using the traditional method. Vehicles can also return empty, but it is more suitable to plan the deliveries in order to avoid this situation, and by means of doing so the use of equipments is optimized.
- **Unloading operation**. In the case of the unloading operation, there is a factor that determines the sequence to carry out. This factor is about if the destination floor of both carts is the same or, by contrast, each cart has a different destination floor. The options are as follows:
  - Both carts go to the same destination floor, which is the origin one. In this case, unloading operations are carried out in opposite order to the loading ones. That is, the towed cart (Number 2) is released when passing through its unloading bay and the vehicle follows to the unloading point of the container that is transported on the platform (Cart 1).
  - The towed cart has its origin point on the same floor but the loaded cart has its one on a different floor. In this case, Cart 2 is released in its unloading bay and the vehicle follows to the destination point of Cart 1 by means of the elevators fitted out for this. In order to make the deliveries’ scheduling and programming tasks easier, we have to take into account that this process order is the most suitable one for this option.
  - Both carts come from floors that are different to the loading ones. In this case, the use of intermediate loading bays is required, which are close to the elevators. When the vehicle arrives at these intermediate loading bays, Cart 2 is released (Fig 12), and the vehicle continues the transport in the elevator to the destination point of Cart 2 (Fig 13). Afterwards, the vehicle returns to the same point to load Cart 2, using the traditional method (Fig 14), and this is transported by means of the elevator to its origin point (Fig 15).

## Material and methods: Design proposal of the required elements

In this section, the design of those main elements which take part at the mechanical level will be developed in more detail, but without reaching their definitive development. This would be like a previous design, in which their main features were defined and which would serve as a work basis for those items of equipment that require a specialized design and would take part in the project. Therefore, the aim is to provide basic information about the main requirements that we have to take into account, and which would serve as an initial database to the prototype and the design stage, following principles of concurrent and collaborative engineering.

### Automated hitching system

**Fig 16:**
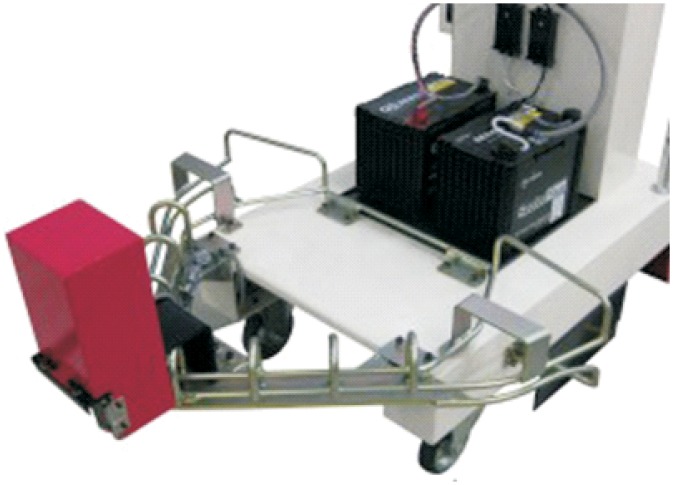
Automated hitching system. Aichi Kikai Techno System Company.

There are some AGV manufacturers for the industrial sector who commercialize units which allow, within the scope of configuration possibilities, to automatically hook carts as an optional accessory [13] [14]. For instance, the Japanese company Aichi Kikai has a device (Fig 16) that can be incorporated to the AGV tow type and that then allows the automated hitch of carts. Its design is optimized to join the tows in the direction of travel of the vehicle, gathering the hitching systems of parked carts when the robot moves forward throughout the predefined route. Later, the device allows the releasing of the gathered cart in another predefined position.

This system has been developed to be applied in tow-type vehicles, but neither in the bibliography nor in the manufacturers’ information has it been proved that these kinds of devices are commercialized with the aim of complementing the unit load carriers type, like those used in hospitals, and that allow them to turn into mixed-type vehicles. This possibility of the incorporation of some optional elements, which is based on modular design [15], would complement the existing equipment, allowing it to implement the system of transport of combined carts, although this implementation would require some modifications in the main equipment in order to be able to join it with the electrical and control systems. By contrast, if the proposed system were developed in a new equipment model, these problems related to the control and electricity systems would be solved more easily, due to this model was already being equipped with the needed requirements in the stage of design [16] [17]. The main elements that are included in the units are as follows:

- **Vehicle’s housing and anchoring system to the vehicle**. This part contains the whole system, which has to be joined to the vehicle and thus makes it possible to drive the cart. Their size must be as small as possible so that this implementation does not modify to a large extent the vehicle’s total length, in order that the vehicles can continue operating in the same conditions when the vehicle doesn’t transport carts by means of this system.
- **Cart’s pivot attachment**. This is the element that is lodged in the cart support and which allows the joining of both parts. This is a moving element which is activated when it detects the cart’s presence in the determined position to hook up both parts.
- **Guides to the hitch**. This element is developed to increase the accuracy in the pivot’s join-up with the support, with the aim of correcting minor deviations that can take place during the hook-up process. As with the previous element, the guides to the hook are a moving element as well, and both are only activated during the hook. Once the hook is carried out, the system lowers, allowing the lateral rotation.
- **Sensor**. This is the element which is responsible for communicating with the equipment’s control system, by means of sending information that allows the autonomy to work the hook unit as much as the main equipment in the new loading conditions.
- **Drive system/motion system**. This element is the one that makes the movement of moving elements possible. This system can be electrical or hydraulic, which are the systems used in the vehicle. By means of the presence of these two different kinds of systems, the unit can share the power source used by the main equipment.
- **Supply system**. This element supplies the needed energy from the vehicle to the system’s functioning. This can be electrical or hydraulic, according to the chosen drive/motion system.

Below is shown a design proposal that includes the previous sections and that can serve as a base for the device’s development.

**Fig 17:**
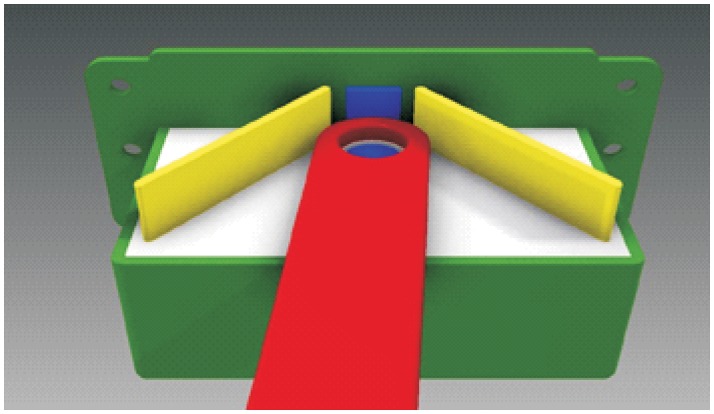
Cross-section view of the hitch in hook-up position.

**Fig 18:**
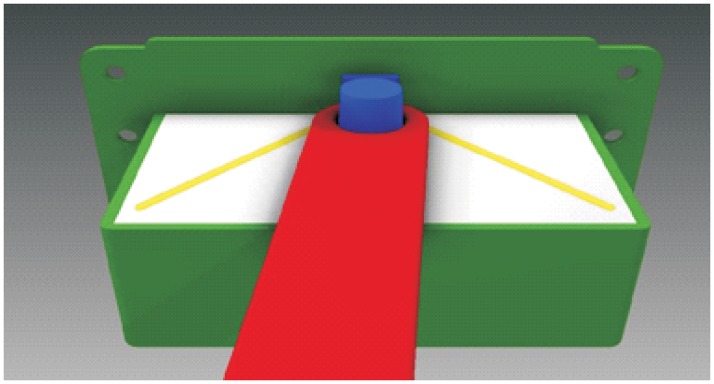
Cross-section view of the hitch coupled.

Fig 17 shows a view of the hitch in the hook-up position. In this case the guides to the hitch are raised, so these direct the support to the exact position to carry out its connection. Once the support is located in this position, this activates the sensor, which is lodged at the bottom, and this allows the raising of the pivot that is lodged in the cavity of the support, and by means of this, both parts are joined. The pivot mentioned has a truncated conical section, with the aim of doing this lodging task more easily. When the pivot is raised lower, the side guides allow the lateral rotation of the unit freely (Fig 18).

**Fig 19:**
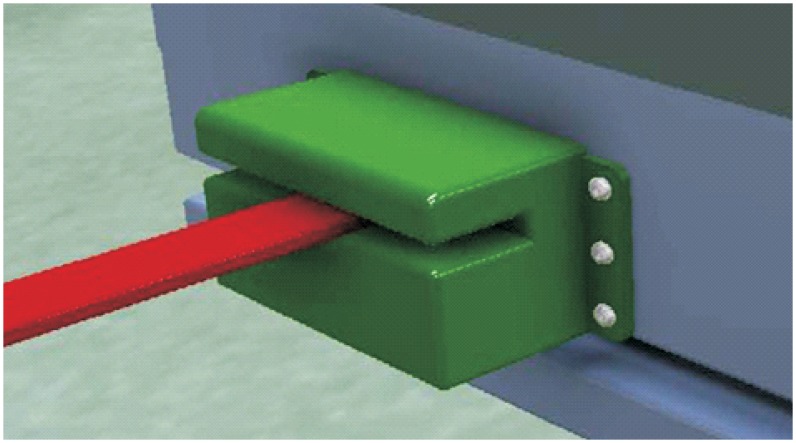
Automated hitching system.

The unit comes with a metal casing, which also serves as an attachment in the case of moving elements. In the bottom part are lodged those mechanical systems that allow the movement of the system and are connected with the equipment’s electrical, hydraulic and control systems. The unit attachment is carried out by means of the inclusion of two flat steel sides; these would join to the cart’s frame by using some nuts and bolts (Fig 19).

### Carts’ joining system

The hitching system must meet two main conditions: that it can be incorporated to the existing carts and that it can be used in both methods of transport (load and tow methods). The proposed system, as a working basis, consists of a folding support which can in turn be driven by the vehicle when this is introduced under the cart to load it, whereas when the cart is in a horizontal position, this is at the right height to be hooked on when the cart is negotiating going backwards. That is, depending on the side from which the vehicle charges the cart, this can be loaded or hooked on. This is a system that functions by means of gravity, thus avoiding the use of electrical or mechanical systems for their activation.

**Fig 20:**
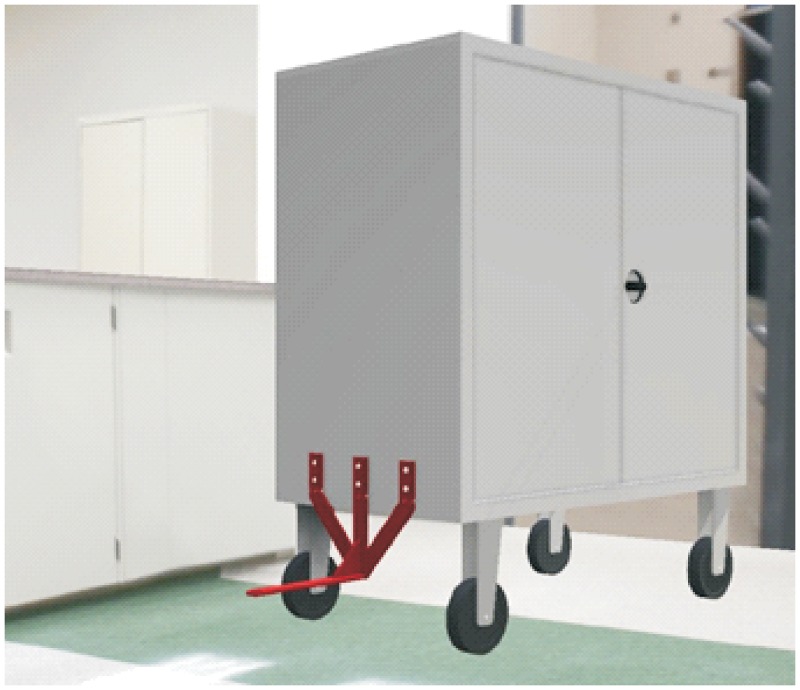
Cart’s view with horizontal hitch (tow).

**Fig 21:**
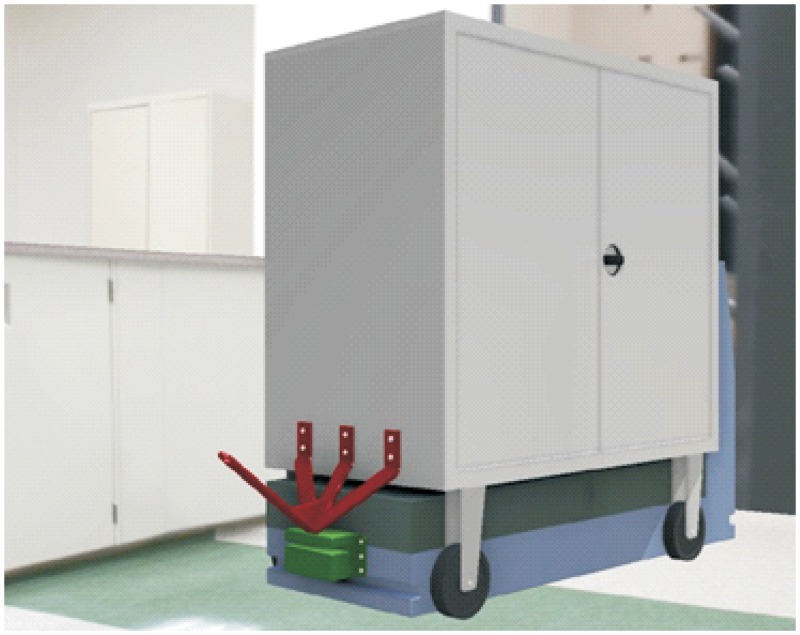
Cart’s view with the hitch folded (load).

The cart’s dimensions with the support folded on the vehicle must not exceed the total length of the vehicle, including the automated hitch unit. In that way, these carts that include the new support can continue working in the spaces they have been assigned and can also use the elevators. Figs 20 and 21 provide a cart’s general view with the system in the two different positions.

**Fig 22:**
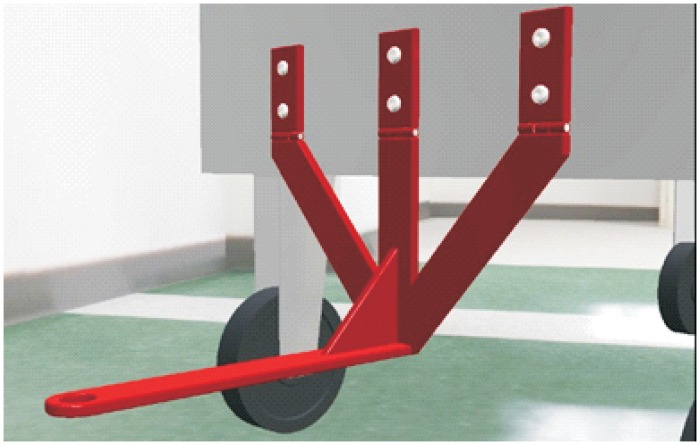
Automated hitching system.

**Fig 23:**
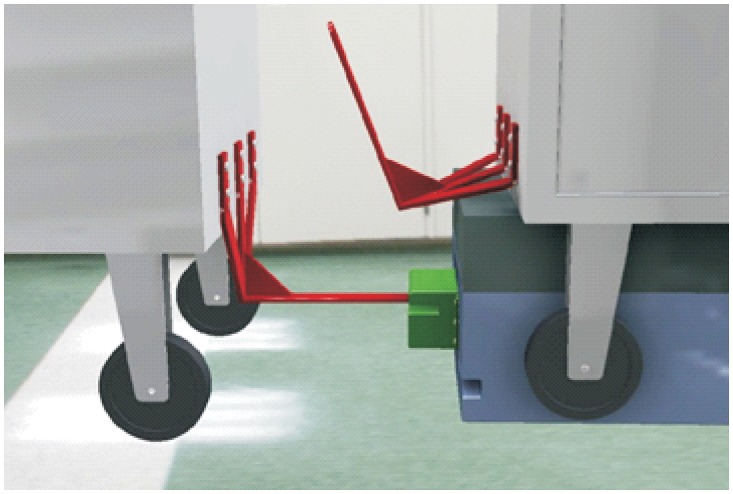
Hitch’s view in both positions.

The unit must be dimensioned in relation to the loadings to which it is exposed, and this must also be made with suitable materials for health purposes. In that sense, the most suitable materials are stainless steel and aluminum, like those used in the cart’s design. The attachment system, which is present inside the cart, must not have a significant effect over the loading space’s dimensions, so that the inner/inside loading capacity is not affected. In the proposed case, this attachment system is reduced to nuts or rivets as well as reinforcing flat steel. Figs 22 and 23 show an expanded view of the proposed system.

The support’s axis of rotation is placed on an elevated position in relation to the underneath surface of the cart, avoiding excessive turning, and at that point is the caisson, which marks the exact position of the vehicle’s hitch when it falls by gravity. Another measure we have to take into account is the placing of a piece made from plastic (material), like a type of nylon, which reduces friction between metal surfaces.

**Fig 24:**
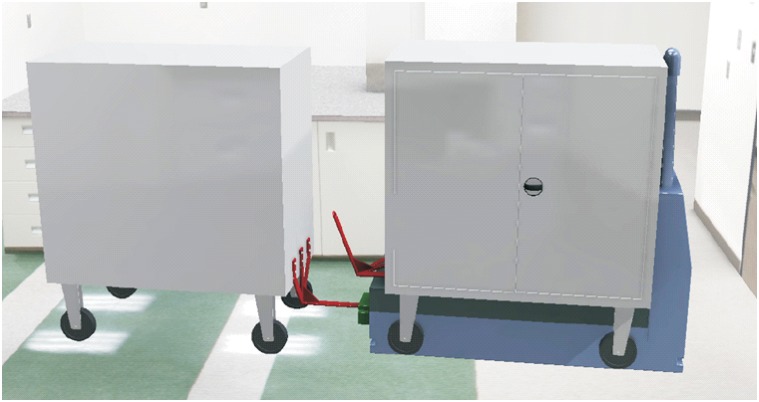
Hitch’s view in both positions.

This piece would be placed in the inner side of the triple support and thus it would contribute to the sliding on the hitch’s chassis and on the vehicle’s platform. In addition to these features, it would prevent possible damages to the material’s surface finish and lower the possibility of emission noises caused by gripping. Fig 24 represents a full towed convoy’s view.

## Results: Analysis of the practical implementation

In order to analyze the scope of application for the optimization proposal, which is laid out throughout the paper, this theoretical impact has been studied in a real environment, specifically in the “Hospital Universitario Río Hortega de Valladolid”, which is a new construction. The hospital was inaugurated in 2009, and a fleet of six automated guided vehicles (AGV) were used from the start in hospital logistics tasks of internal distribution [18].

The results have been obtained with an analytical simulator, and it has been thanks to the results obtained in this simulation that the project has obtained a favorable report and will be implemented when funds are available

**Fig 25:**
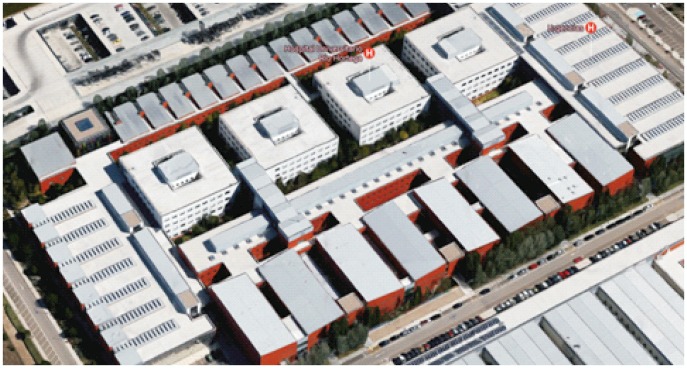
General view of Hospital Río.

This hospital is divided into different blocks, differentiated by their function and their use (Fig 25), having a large floor extension. On the one side there is the industrial area, which is the point of origin of materials to transport, and on the other side there are the units, which are the destination points. This building has a large extended floor and also has four vertical communication hubs, including specific elevators to be used by automated guided vehicles.

There are many tasks assigned to robots, which have an almost full operational capability. Those materials that are transported by these vehicles and are arranged in carts are mainly: meal trays, linen and uniforms, and supplies and materials for medical use. The programming and the kind of software used in AGVs do not assign predetermined tasks to each vehicle, but these tasks are delivered between the operative vehicles in each moment, depending on their closeness or their availability. Below is the analysis of the optimization proposal’s repercussion in a specific service: the delivery of linen in carts. This is the delivery of linen which is used in the inpatient units and whose quantity and scheduling deliveries have been set previously. Nowadays, the delivery is carried out on the graveyard shift and these have destination units which are distributed all over the hospital and all the floors. In order to do this, it is necessary to use the four elevator units that do this specific task to represent the whole study.

**Fig 26:**
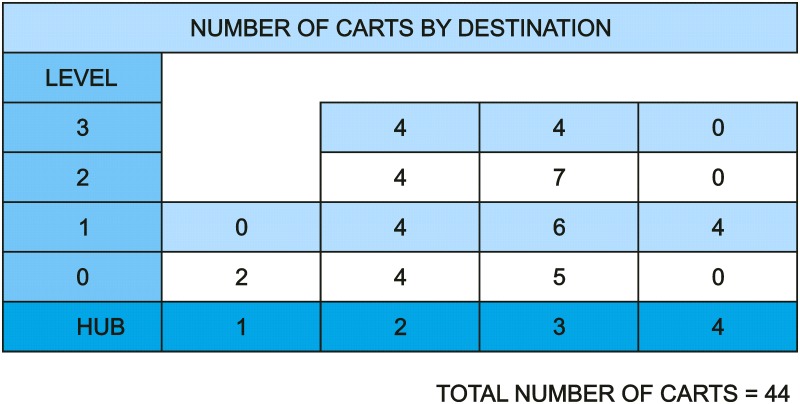
Carts by destination.

Thus, the conclusion extracted from this study can be extrapolated to the whole set of tasks carried out by these vehicles. The number of carts that are sent to each point of consumption is shown in Fig 26. The destination units are organized by floor and in function of the communication hub they use. Unloading is carried out in the equipped bays close to the elevators and later it is carried out in the final delivery to each specific destination manually.

**Fig 27:**
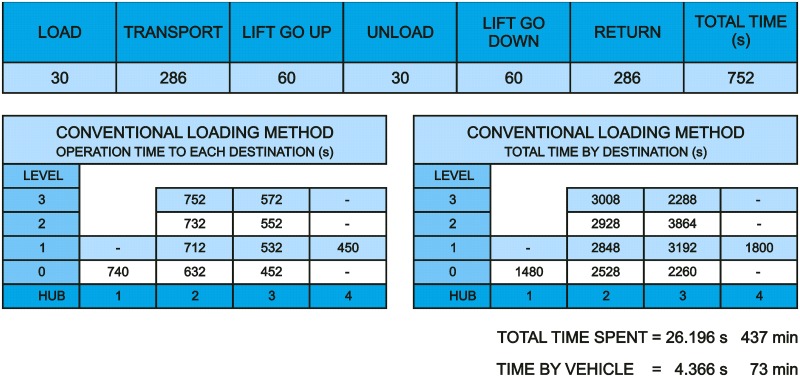
Data from conventional loading method.

Below, in Fig 27, are shown the times spent on each operation and the total times spent on the whole process when the current loading method with a single cart in each shift to the destination points is used. Times spent on each operation are not fixed because these depend on the elevator availability; this is why the average use times have been taken. The times have been measured from loading and return point to the same origin point so that it can be possible to connect successive operations. In Fig 27, it is also reflected, measured in seconds, the sequence of operations to do, taking Level 3-Hub 3 as an example.

**Fig 28:**
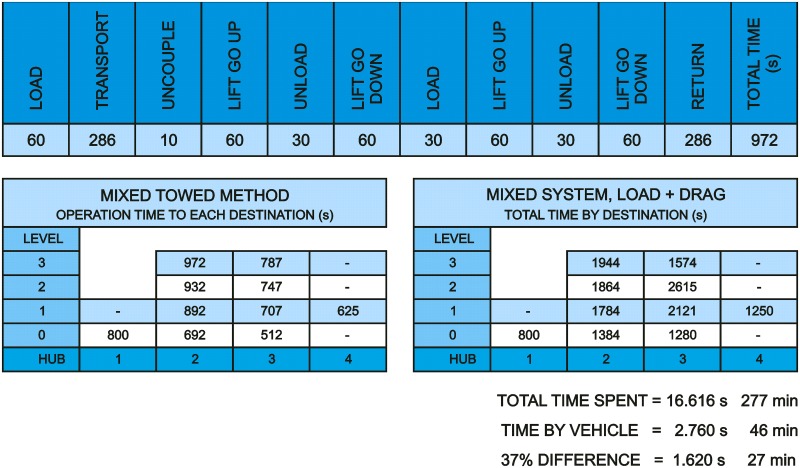
Data from the proposed mixed towing method.

Fig 28 shows times spent with the mixed system of combined carts; in the first place time spent on each operation is analyzed, and in the second place the total time spent on the whole process is analyzed. In this analysis, in order to achieve homogeneous and comparable results between themselves, the same criteria have been taken into account as regards average times marked by elevators’ availability. Fig 28 shows the sequence of operations, which in this case is expressed in seconds, and is specified by Level 3-Hub 3.

On the basis of the obtained data from the study that is developed throughout this paper, it can be concluded that: by means of the implementation of this method, which is based on the transport of combined carts, in the delivery of linen carts a 36.57% reduction in time in relation to time spent using the traditional method can be achieved. This means a time reduction of 26.61 minutes by each one of the six vehicles. Or, in a more representative way: four vehicles would be able to carry out the delivery tasks in the same time that, currently, is taken by the six available units.

## Conclusions

Throughout this paper, the necessary requirements have been analyzed, in succession, in order to allow the development of the optimization proposal that has been put forward. These requirements have been tackled from the different points of view needed to ensure that the measure’s implementation functions properly. First, these requirements have been analyzed from a general overview, and then all the elements that are involved in the process have been developed in a more specific way. Finally, the effect that the measure’s implementation would have in a specific place and using real data in order to establish a comparison with the system’s potential advantages has been studied.

We consider that this is an innovative proposal that tries to combine the advantages of the two main methods of transport used by automated guided vehicles (AGVs), which are towing method and loading method. By means of the combination of these two methods, a mixed method that can provide a significant reduction of time used to carry out the tasks assigned to vehicles is achieved. Specifically, in the study that was carried out according to real data from the Hospital Universitario Rio Hortega (Valladolid), it was seen that it can achieve a reduction of over 35% regarding total time used currently. This means that the studied task would be able to be carried out by four vehicles in the same time that is currently carried out using a fleet that consists of six units. This adjustment can turn into a reduction of the numbers of equipment, with a unit cost above €90.000, or this can also contribute to the assignment of new tasks to those vehicles that are released after the implementation of the new delivery system.

In this report it has been shown that the implementation of this optimization proposal can be resolved by means of applying specific solutions at operational level, and also in the field of development of mechanical elements. However, the modification of the equipment’s configuration requires as much of a differential treatment as the modification of the management software because of the fact that, in great measure, control systems are closed, which implies certain limitations due to software architecture, and their modification would be not possible without the direct participation of equipment manufacturers. Therefore, the challenge is to develop control systems which are more open than the current ones are, in order to make their configuration for the users attending to their needs easier. This, coupled with improvements in the mechanical and operational design, would mean that it would be able to improve the equipment’s performance, which supposes an even greater increase in the possibilities for extending the use of these kinds of vehicles.
